# Quantitative histological models suggest endothermy in plesiosaurs

**DOI:** 10.7717/peerj.4955

**Published:** 2018-06-06

**Authors:** Corinna V. Fleischle, Tanja Wintrich, P. Martin Sander

**Affiliations:** 1 Steinmann-Institut für Geologie, Mineralogie und Paläontologie, Rheinische Friedrich-Wilhelms-Universität Bonn, Bonn, Germany; 2 Dinosaur Institute, Natural History Museum of Los Angeles County, Los Angeles, USA

**Keywords:** Bone histology, Plesiosauria, Vascular density, Phylogenetic eigenvector maps, Metabolism, Bone growth rate, Phylogenetic comparative methods, Marine reptile, Endothermy

## Abstract

**Background:**

Plesiosaurs are marine reptiles that arose in the Late Triassic and survived to the Late Cretaceous. They have a unique and uniform bauplan and are known for their very long neck and hydrofoil-like flippers. Plesiosaurs are among the most successful vertebrate clades in Earth’s history. Based on bone mass decrease and cosmopolitan distribution, both of which affect lifestyle, indications of parental care, and oxygen isotope analyses, evidence for endothermy in plesiosaurs has accumulated. Recent bone histological investigations also provide evidence of fast growth and elevated metabolic rates. However, quantitative estimations of metabolic rates and bone growth rates in plesiosaurs have not been attempted before.

**Methods:**

Phylogenetic eigenvector maps is a method for estimating trait values from a predictor variable while taking into account phylogenetic relationships. As predictor variable, this study employs vascular density, measured in bone histological sections of fossil eosauropterygians and extant comparative taxa. We quantified vascular density as primary osteon density, thus, the proportion of vascular area (including lamellar infillings of primary osteons) to total bone area. Our response variables are bone growth rate (expressed as local bone apposition rate) and resting metabolic rate (RMR).

**Results:**

Our models reveal bone growth rates and RMRs for plesiosaurs that are in the range of birds, suggesting that plesiosaurs were endotherm. Even for basal eosauropterygians we estimate values in the range of mammals or higher.

**Discussion:**

Our models are influenced by the availability of comparative data, which are lacking for large marine amniotes, potentially skewing our results. However, our statistically robust inference of fast growth and fast metabolism is in accordance with other evidence for plesiosaurian endothermy. Endothermy may explain the success of plesiosaurs consisting in their survival of the end-Triassic extinction event and their global radiation and dispersal.

## Introduction

As marine reptiles, plesiosaurs and their aquatic adaptations have aroused interest in many different ways, including locomotion with hydrofoil-like flippers (underwater flight), evolution of a long neck, reproduction, and viviparity (O’Keefe & Chiappe, 2011; Wintrich et al., 2017). Plesiosaur morphology is both unique among all known taxa that ever lived on Earth and strikingly uniform. It has long been assumed that plesiosaurs evolved in the earliest Jurassic (Kelley et al., 2014; Bardet et al., 2014) and died out at the end of the Cretaceous (Rieppel, 2000; Motani, 2009). The discovery of *Rhaeticosaurus* from the Rhaetian of Germany (Wintrich et al., 2017) confirms earlier suggestions, however, of a radiation of plesiosaurs in the Late Triassic (Benson, Evans & Druckenmiller, 2012). Subsequently, plesiosaurs rapidly spread around the globe (Benson, Evans & Druckenmiller, 2012; Benson & Druckenmiller, 2014; Wintrich et al., 2017). Until today, the basis of their success remains poorly understood.

Plesiosauria belong to the clade Sauropterygia which diversified in the Triassic into the Placodontia and the Eosauropterygia. These, in turn, include the possibly paraphyletic pachypleurosaurs and the Eusauropterygia, which are divided into Nothosauroidea and Pistosauroidea, the line including some stem taxa, the pistosaurs, and the Plesiosauria. All non-plesiosaur sauropterygians went extinct at the end of the Triassic.

### Endothermy

It is widely agreed that endothermy evolved several times independently. Among recent species, true endothermy, however, is only present in mammals and birds. The evolutionary origin and development of endothermy and metabolism presents a special challenge and has always been a matter of debate (Nespolo et al., 2011).

Endothermic animals are able to generate heat in order to raise and keep their body temperature at a stable and high level, independently from ambient temperatures. Ectotherms, on the other hand, depend upon their thermal environment (Hickman et al., 2001). The evolution of endothermy includes the ability to maintain a high body temperature and a high basal metabolic rate (Crompton, Taylor & Jagger, 1978). The relationship between endothermy and metabolic rate (usually measured as oxygen consumption per unit time) has long been recognized and is based on the energetic costs of heat production (Nespolo et al., 2011). The active lifestyle of endotherms enables organisms to live in unfavorable environments, opening niches unavailable to ectotherms. As a consequence, endotherms suffer from less competition and even have higher reproductive rates (Koteja, 2004). However, to maintain a high body temperature, they have a high energy demand and require constant food uptake, which is again only possible by a high level of activity. Metabolic rate is then related to growth rate, as only a high metabolism may provide enough energy for fast growth, a connection that has been proven statistically (Montes et al., 2007).

### Plesiosaur metabolism

In plesiosaurs, the sauropterygian crown-group, several findings point towards a high growth rate and an elevated metabolism. One feature regularly encountered in plesiosaur long bone epiphyses is small pits on the external surface, which correspond to large vascular canals inside the bone running longitudinally from the middle of the bone through the cartilage cap (Moodie, 1916; Fostowicz-Frelik & Gazdzicki, 2001; Liebe & Hurum, 2012). This pit structure had been detected in the leatherback turtle (*Dermochelys coriacea*) (Rhodin, 1985). Due to the connection of the pits to vascularization, it has been hypothesized that they are an indicator for rapid growth in these turtles and plesiosaurs (Rhodin, 1985; Liebe & Hurum, 2012). Independent evidence for an active lifestyle in the open sea is found in paleobiogeography, as plesiosaurs are recorded globally from marine open-water deposits (Benson, Evans & Druckenmiller, 2012; Benson & Druckenmiller, 2014; Wintrich et al., 2017). However, several prerequisites must be fulfilled in order to achieve such a pelagic lifestyle. From studies on Triassic sauropterygians including nothosaurs and pistosaurids and a relationship between bone mass decrease (bmd) and body size, Krahl, Klein & Sander (2013) conclude that a general increase in body size, as it is also found in plesiosaurs, and high growth rates are required to become an open-water cruiser. For active swimming, furthermore, a high metabolic rate is considered necessary, also regarding a predatory diet (Druckenmiller & Russell, 2008) and a certain inevitable cold water resistance (Klein, 2010; Houssaye, 2013; Krahl, Klein & Sander, 2013). The latter is supported by fossils from the Cretaceous of Australia and Antarctica, where near freezing water temperatures are thought to have prevailed (Kear, 2006; O’Gorman, Talevi & Fernandez, 2017; Ossa-Fuentes, Otero & Rubilar-Rogers, 2017). Also, dispersal before the breakup of Pangea and establishment of an equatorial seaway in the Late Jurassic would have had to involve polar regions (Bardet et al., 2014).

Assuming a high growth rate in plesiosaurs on aforementioned grounds and an enormous increase in body size of juveniles in their first year, seen in growth marks and bone apposition (Wintrich et al., 2017), a vertical energy transfer from mother to calf is necessitated. Indeed, a gravid specimen demonstrates viviparity and large, single progeny, suggesting a K-selected reproductive strategy (O’Keefe & Chiappe, 2011). Providing enough food to supply the young for fast growth requires extensive hunting and thus fast, sustained swimming and may hence argue for endothermy.

Furthermore, geochemical analyses of oxygen isotopes in plesiosaur teeth argue in favor of maintaining a stable high body temperature in tropical and cold temperate environments. Estimated body temperatures were as high as 35 ± 2 °C (Bernard et al., 2010).

Further evidence comes from bone histology, where Wintrich et al. (2017) inferred the evolution of fast growth and elevated metabolic rate based on qualitative histological indicators and quantitative comparisons with endothermic animals.

### Endothermy and plesiosaurian bone histology

Plesiosaur histology has been studied for far more than 100 years (Kiprijanoff, 1881; Wiffen et al., 1995; Fostowicz-Frelik & Gazdzicki, 2001; Salgado, Fernandez & Talevi, 2007; Liebe & Hurum, 2012; Wintrich et al., 2017). However, their bone histology remains insufficiently studied in contrast to basal sauropterygians (Klein, 2010; Houssaye, 2013; Krahl, Klein & Sander, 2013; Houssaye, Sander & Klein, 2016; Klein et al., 2016). Here, we concentrate on the midshaft histology of stylopodial bones (humerus, femur), which commonly cannot be distinguished morphologically in plesiosaurs. Young adult specimens show a very small medullary cavity and full preservation of the growth record (Wintrich et al., 2017). In older specimens, primary bone has been replaced by dense Haversian bone (Kiprijanoff, 1881; de Buffrénil & Mazin, 1992; Wiffen et al., 1995; Krahl, Klein & Sander, 2013; Wintrich et al., 2017). In the past, ontogenetic stage and also plane of sectioning had prevented the detection of uniformity and uniqueness of plesiosaurian bone histology (Wintrich et al., 2017).

In amniote cortical bone histology, the principle that bone tissue type reflects bone growth rate is known as “Amprino’s rule” (Amprino, 1947). One of these bone tissue types is fibrolamellar bone (FLB). FLB consists of a fast growing scaffold of unorganized woven bone matrix with highly organized lamellar bone infilling the numerous primary vascular canals and forming primary osteons (de Ricqlès, 1974; Stein & Prondvai, 2014). Among extant amniotes, FLB is only found in mammals and birds, suggesting a link to high growth rates (Padian, de Ricqlès & Horner, 2001; Cubo et al., 2012; Houssaye, 2013; Padian & Lamm, 2013; Stein & Prondvai, 2014).

Quantitative statistical analyses of primary osteons, one feature of FLB, demonstrate that the presence and proportion of primary osteons, hence the degree of vascularization, is directly connected to bone growth rate (de Margerie, Cubo & Castanet, 2002). Beyond that, theoretically, strong vascularization is also related to a high metabolic rate, as an elevated oxygen consumption leads to higher metabolic demands and necessitates oxygen supply (Bennett & Ruben, 1979; Montes, Castanet & Cubo, 2010). This is supported by the idea that bone growth rate correlates with metabolic rate, as only a high metabolism may provide materials and energy for fast growth (Wintrich et al., 2017). A positive linear relationship between log10 periosteal bone growth rate and log10 geometry-corrected resting metabolic rate (RMR) in extant growing amniotes provides statistical support and the possibility to infer metabolic rate from bone growth rate (Montes et al., 2007). Furthermore, vascular canal orientation may indicate bone growth rate, with radial canals being the fastest deposited ones (de Ricqlès, Padian & Horner, 2003; de Margerie et al., 2004; Klein et al., 2016). However, also a biomechanical explanation for canal orientation must be considered (de Margerie, Cubo & Castanet, 2002). For plesiosaurs, Wintrich et al. (2017) described primary periosteal compact bone of well-vascularized FLB with (not strict) radial canals, hereby indicating fast growth.

Besides vascularity, cellular characteristics may be informative for bone growth and metabolism. Osteocytes formed by static ossification are rather large and plump, irregularly shaped, and randomly arranged. Since they are present in woven bone, they indicate fast bone deposition (Palumbo, Ferretti & Marotti, 2004; Marotti, 2010; Stein & Prondvai, 2014). The presence of osteocytes formed by static ossification has been described for plesiosaurs (Wintrich et al., 2017).

Since all these findings in histology point towards endothermy in plesiosaurs, this study performs quantitative estimates in a phylogenetic comparative framework for both metabolic rate and bone growth rate.

### Phylogenetic comparative methods in the study of bone histology

According to Seilacher (1970), any characteristic of an organism is the outcome of shared ancestry, function, and structural properties. For bone growth rate, it was shown that all three components of “Seilacher’s triangle” partially serve as explanatory variables (Cubo et al., 2008). Also for bone vascularity, phylogeny, apart from growth rate, explains significant portions of variation (Montes, Castanet & Cubo, 2010). These findings demonstrate the necessity for phylogenetic corrections when estimating growth rate or related variables from bone histology. Qualitative and quantitative studies of bone tissue types and the meaning for bone growth rate (de Buffrénil & Pascal, 1984; Castanet, Francillon-Vieillot & Bruce, 1996, Castanet et al., 2000; de Margerie et al., 2004; de Ricqlès et al., 2008) were finally followed by statistical analyses incorporating the influence of shared ancestry, so-called phylogenetic comparative methods (PCM) (Cubo et al., 2012; Legendre, Segalen & Cubo, 2013).

During the last decade, the popularity of PCM in paleontology has increased significantly. Recently, some studies thereby focused on estimating physiological parameters in fossils. An alternative to the common method of phylogenetic generalized least squares (Organ et al., 2007, 2011; Organ, Brusatte & Stein, 2009; Huttenlocker & Farmer, 2017) is phylogenetic eigenvector regression, a method developed by Diniz-Filho, de Sant’ana & Bini (1998). After some criticism due to the lack of an included evolutionary model (Adams & Church, 2011; Freckleton, Cooper & Jetz, 2011), Guénard, Legendre & Peres-Neto (2013) proposed an alternative method called phylogenetic eigenvector maps (PEM), which has been recently used to estimate parameters in fossils, taking into account both phylogeny and phenotypic variables (Legendre et al., 2016; Olivier et al., 2017).

## Materials and Methods

### Material

**Figure 1:**
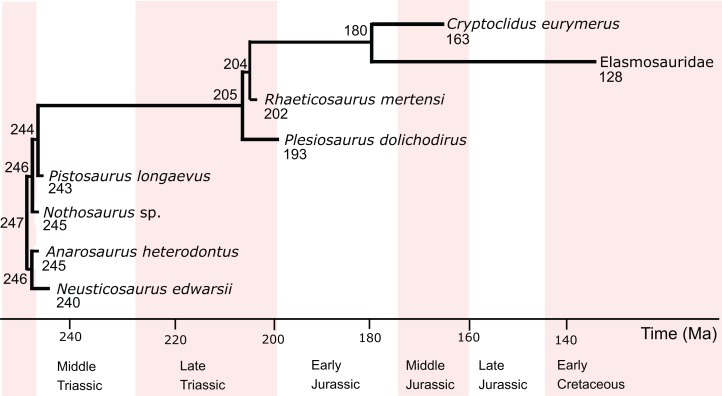
Phylogenetic relationships, divergence times, and ages of the eosauropterygians investigated in this study. The information is taken from Rieppel (2000), Ketchum & Benson (2010), and Wintrich et al. (2017). Divergence times (in Ma) are indicated at the nodes, and tip ages (in Ma) are indicated below the genus names.

The two predictive models constructed in this study include eight eosauropterygians (Table 1), including pachypleurosaurs (*Anarosaurus heterodontus*, *Neusticosaurus edwarsii*), the nothosauroid *Nothosaurus* sp., the pistosaur *Pistosaurus longaevus*, and the plesiosaurs *Plesiosaurus dolichodirus*, *Rhaeticosaurus mertensi*, *Cryptoclidus eurymerus* and an indeterminate elasmosaur from Japan, hitherto referred to as Elasmosauridae (Fig. 1). Standard petrographic thin sections of 50–80 μm thickness from long bone mid-diaphyses were investigated (Fig. S1), all of which were the subject of earlier studies (Sander, 1989, 1990; Klein, 2010, 2012; Krahl, Klein & Sander, 2013; Wintrich et al., 2017). Overview images were taken with an Epson V750 scanner. The sections were further investigated at higher magnification using a Leica DM2500LP polarizing microscope, and images were taken with a Leica DFC420 color camera mounted on this microscope and the software EASYLAB 7.

**Table 1:** Overview and supplementary information about the analyzed eosauropterygian specimens.

Species	Higher taxon	Specimen number	Bone	Plane of section	Previous studies	Geological time	Reference to geological time
*Anarosaurus heterodontus*	Pachypleurosauridae	Wijk. 06-38fe	Femur	Transverse	Klein (2012)	Middle Triassic	Klein (2012)
*Neusticosaurus edwardsii*	Pachypleurosauridae	PIMUZ T3455	Humerus	Transverse	Sander (1989, 1990)	Middle Triassic	Carroll & Gaskill (1985)
*Nothosaurus* sp.	Nothosauroidea	IGWH 21	Femur	Transverse	Klein (2010)	Middle Triassic	Rieppel (2000) and Rieppel & Wild (1996)
*Pistosaurus longaevus*	Pistosauroidea	SMNS 84825	Humerus	Transverse	Krahl, Klein & Sander (2013)	Middle Triassic	Geissler (1895) and Sues (1987)
*Plesiosaurus dolichodirus*	Plesiosauria	STIPB R90	Femur	Transverse	Wintrich et al. (2017)	Early Jurassic	Conybeare (1824)
*Rhaeticosaurus mertensi*	Plesiosauria	LWL-MfN P 64047 section PM 3	Femur	Transverse	Wintrich et al. (2017)	Late Triassic	Wintrich et al. (2017)
*Cryptoclidus eurymerus*	Plesiosauria	STIPB R 324	Femur	Transverse	Wintrich et al. (2017)	Middle Jurassic	Brown & Cruickshank (1994)
Elasmosauridae indet.	Plesiosauria	OMNH MV 85	Humerus	Transverse	Wintrich et al. (2017)	Late Cretaceous	Williston (1906)

**Note:**

Abbreviations of collection numbers: Wijk, National Museum of Natural History Naturalis, Leiden, The Netherlands; PIMUZ, Paleontological Institute and Museum of the University of Zurich, Switzerland; IGWH, Institute of Geosciences of the Martin-Luther-University Halle-Wittenberg, Germany; SMNS, Stuttgart State Museum of Natural History, Germany; STIPB, Steinmann-Institute, Division of Paleontology, University of Bonn, Germany; LWL-MfN, Landschaftsverband Westfalen-Lippe, Museum für Naturkunde, Münster, Germany; OMNH, Osaka Museum of Natural History, Osaka, Japan. Histological thin sections are housed in the histology slide collections of the Steinmann Institute, University of Bonn, Germany.

### Phylogenetic eigenvector maps

The R package MPSEM (Guénard, Legendre & Peres-Neto, 2013) was used to convert the phylogenies into PEMs and build predictive models to estimate the response variable (metabolic rate, growth rate) for eosauropterygians. Besides phylogeny, one additional variable (primary osteon density) was taken into account for each prediction. Although in theory, more predictor variables may be included, this is a potential source of error considering the small sample size (Mundry, 2014). Both predictor and response variables were log transformed to accommodate the large range of values in the data set. When several alternative predictor variables are provided, the best model was selected based on the Akaike information criterion, corrected for small sample size (Burnham, Anderson & Huyvaert, 2011) and cross-validated using leave-one-out cross-validation, suitable to a small training data set. The response variable was then estimated for each fossil specimen with 95% confidence intervals. After having estimated the response variable for sauropterygians, ancestral states for the complete sample set were reconstructed and plotted color-coded on the respective phylogenetic tree, using the R package phytools (Revell, 2012), following the approach of Legendre et al. (2016).

### Phylogenetic hypotheses

The initial phylogenetic relationships of extant taxa for the different analyses are taken from the respective studies where the response variables had been determined: Bone growth rate: Cubo et al. (2012); RMR: Legendre et al. (2016). Fossils other than those studied here were not added to the phylogenetic hypotheses, even if one of the parameters had been inferred for these in the past. Turtles were added as the sister group to archosaurs in all analyses of this study. The phylogenetic position of turtles is controversial. While most morphological paleontological analyses argue in favor of a close relationship of turtles and lepidosaurs (Zardoya & Meyer, 2001; Schoch & Sues, 2015), most molecular studies support a close relationship of turtles and archosaurs (Iwabe et al., 2004; Roos, Aggarwal & Janke, 2007; Chiari et al., 2012; Crawford et al., 2012). Finally, the fossil eosauropterygians were added to the trees of extant species. The origin and ancestry of sauropterygians is unknown so far, however, a sister group relationship with Lepidosauria is widely accepted (Rieppel, 2000; Chen et al., 2014).

For internal eosauropterygian relationships, the classical view of pachypleurosaurs as sister group to eusauropterygians including Nothosauroidea and Pistosauroidea was chosen (Rieppel, 2000). The ingroup relationships of Pistosauroidea were taken from Ketchum & Benson (2010) and Wintrich et al. (2017) (Fig. 1). For the analyses, all branch lengths were rounded to the nearest integer.

### Predictor and response variables

As additional predicting variable for both statistical models, primary osteon density (vascularity) was chosen. Bone histological measurements were taken in the area of sustained high growth in the inner cortex before the first growth mark. The area of sustained high growth is located around the inflection point between the acceleration and the deceleration growth phase in modeled sigmoid growth curves (Cubo et al., 2012). To compensate for possible mechanical stress-related structural differences, measurements were taken in four areas in the cortex using Fiji/ImageJ (Version 1.51n) (Schindelin et al., 2012).

**Figure 2:**
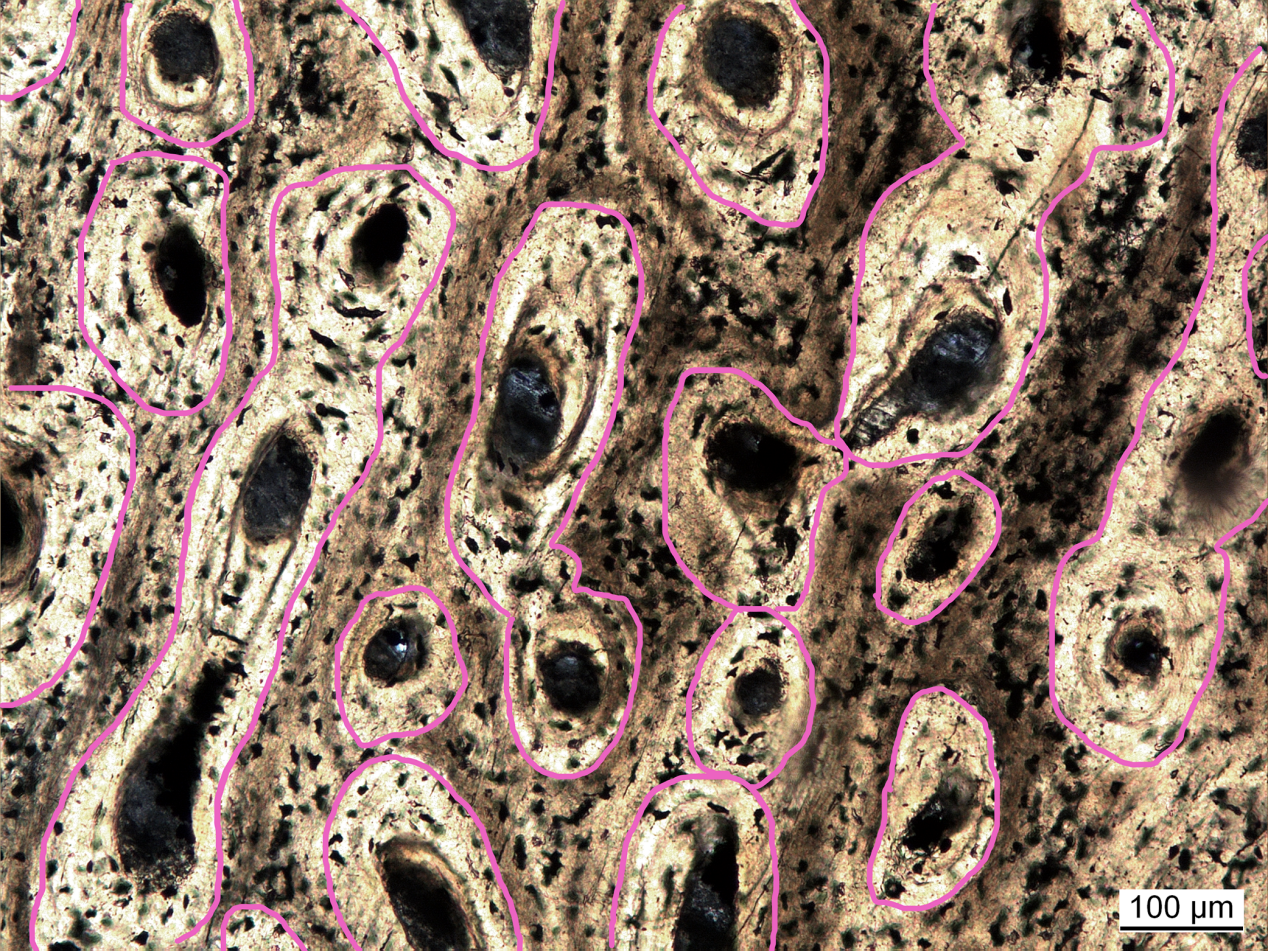
Measuring primary osteon density exemplified for *Plesiosaurus dolichodirus*. Primary osteon density is measured as the (pink circled) area of the vascular canals with the infillings by lamellar bone (primary osteons) compared to total bone area. Photo by Corinna Fleischle.

Vascular density had initially been measured as the ratio of vascular area (in simple vascular canals) to total primary bone area (Cubo et al., 2005). In follow-up studies, vascular density was quantified as the number of canals divided by total bone area (Cubo et al., 2012; Legendre et al., 2016). In these studies it was argued that the sampled extant taxa were still growing and, subsequently, osteons were not yet infilled, complicating comparisons to fossil taxa with infilled canals. However, the method using canal number seems biased by the canal orientation. Bones with canals running parallel to the cutting plane of the cross section will always exhibit a lower number of canals per area and thus will have lower vascular density values compared to bones with longitudinal canals. To avoid this bias, a new approach is proposed here. Inspired by Cubo et al. (2005), vascular density is again defined as the proportion of vascular area to whole bone area. However, to ensure comparability between simple canals and filled osteons, the areas of the latter are measured incorporating the lamellar bone making up the infillings (Fig. 2), which are mostly unambiguously visible. With this approach, vascular density (primary osteon density) was measured in extinct sauropterygians (Table S2) and reanalyzed for extant species from previous studies (Cubo et al., 2012; Legendre et al., 2016) either directly under the microscope or in high resolution images (Table S1).

The response variables in extant species (metabolism and growth rate) were taken from the respective published literature. Values for mass-specific RMR, measured in ml O_2_ h^−1^ g^−0.67^, were taken from Legendre et al. (2016). Values for periosteal bone growth rate as quantified from fluorescent labeling were taken from Cubo et al. (2012).

## Results

**Figure 3:**
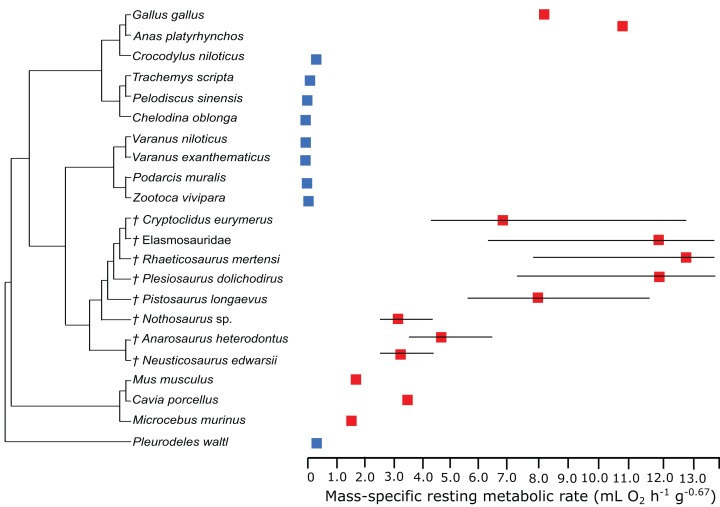
Measured and estimated mass-specific resting metabolic rate (RMR) with 95% confidence intervals. Blue boxes indicate ectothermic species, red boxes indicate (possibly) endothermic species. All sauropterygians in the model have a RMR higher than the analyzed ectothermic species. For plesiosaurs, a RMR in the range of birds was even estimated.

**Figure 4:**
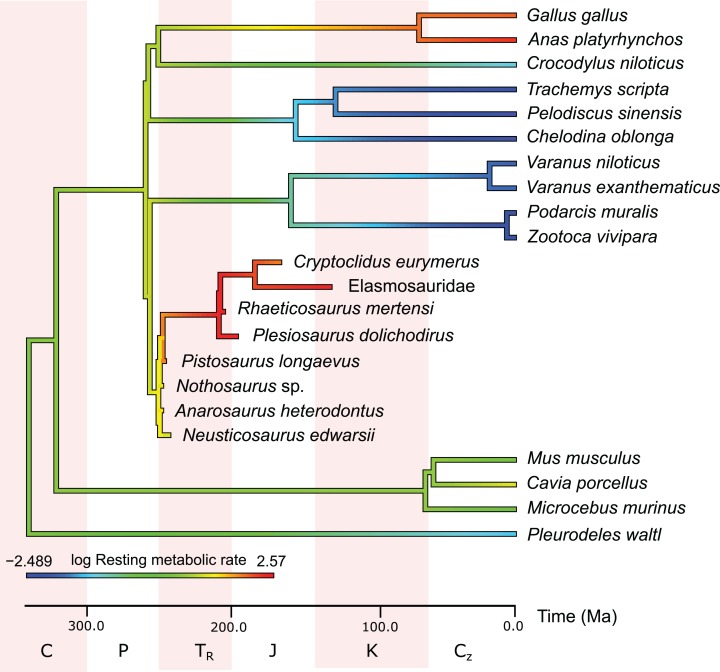
Measured and estimated mass-specific resting metabolic rate (RMR) in log mL O_2_ h^−1^ g^−0.67^ mapped color-coded on a phylogenetic tree. In this comparison, blue represents low values, green and yellow intermediate values and red high values. Plesiosaurs have a RMR in the range of birds. Within sauropterygians, the RMR increases, but even basal forms show values in the range of mammals or higher and far above the ectothermic species included in the model.

In the PEM for metabolic rate (for the model and its coefficients see the supplementary information), the lowest values in sauropterygians were obtained for *Nothosaurus* (3.1 mL O_2_ h^−1^ g^−0.67^), *Neusticosaurus* (3.2 mL O_2_ h^−1^ g^−0.67^), and *Anarosaurus* (4.8 mL O_2_ h^−1^ g^−0.67^). Higher values were predicted for *Cryptoclidus* (7.2 mL O_2_ h^−1^ g^−0.67^) and *Pistosaurus* (8.033 mL O_2_ h^−1^ g^−0.67^). Highest values were estimated for *Plesiosaurus* (12.0 mL O_2_ h^−1^ g^−0.67^), the elasmosaur (12.1 mL O_2_ h^−1^ g^−0.67^), and *Rhaeticosaurus* (13.1 mL O_2_ h^−1^ g^−0.67^). The estimated values show the evolution of the RMR from basal eosauropterygians towards plesiosaurs. Before the evolution of plesiosaurs, the RMR increases. Figure 3 shows measured and estimated RMR with 95% confidence intervals (for exact values see Table S4). In Fig. 4, measured RMR of extant taxa and estimated RMR of sauropterygians are plotted on the phylogenetic tree.

**Figure 5:**
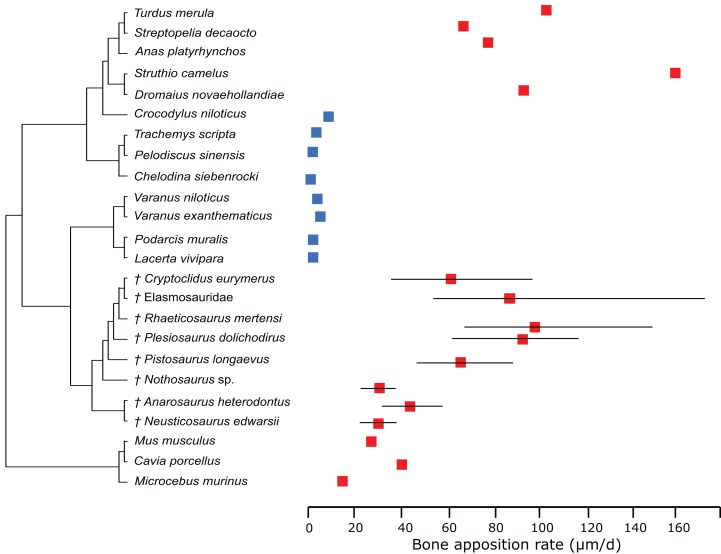
Measured and estimated bone apposition rate with 95% confidence intervals. Blue boxes indicate ectothermic species, red boxes indicate (possibly) endothermic species. All sauropterygians show a growth rate higher than the analyzed ectothermic species. Plesiosaurs have values in the range of birds.

**Figure 6:**
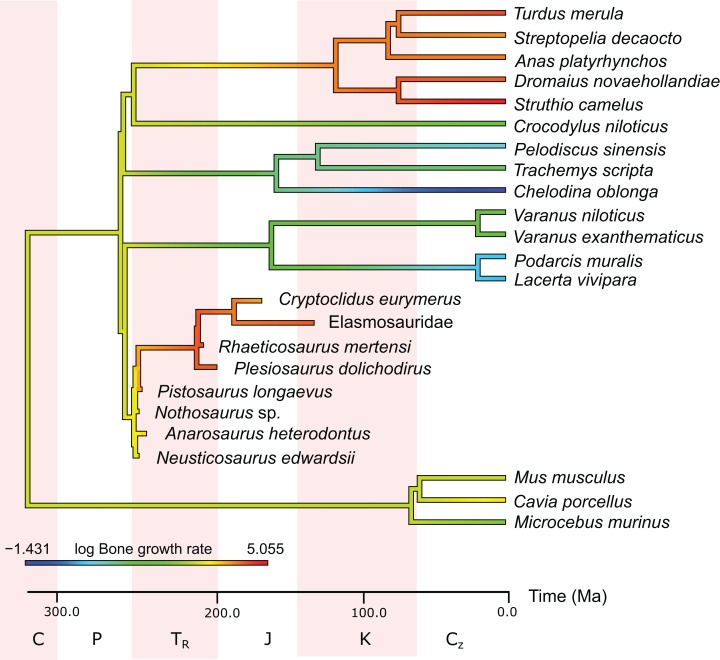
Measured and estimated bone apposition rate in log μm/day mapped color-coded on a phylogenetic tree. In this comparison, blue represents low values, green and yellow intermediate values and red high values. Plesiosaurs have a growth rate in the range of birds. Within sauropterygians, growth rate increases, but even basal forms show values in the range of mammals or higher and far above the ectothermic species included in the model.

In the PEM analysis for bone growth rate (for the model and coefficients see the supplementary information), the lowest values were estimated for *Nothosaurus* (29.4 μm/day), *Neusticosaurus* (29.8 μm/day), and *Anarosaurus* (42.4 μm/day). Higher values were predicted for *Cryptoclidus* (60.0 μm/day) and *Pistosaurus* (65.7 μm/day). Highest bone growth rates were estimated for *Plesiosaurus* (92.9 μm/day), the elasmosaur (93.6 μm/day), and *Rhaeticosaurus* (99.7 μm/day). The estimated values show that bone growth rate increases before the evolution of plesiosaurs. Figure 5 shows measured and estimated growth rate with 95% confidence intervals (for exact values see Table S6). In Fig. 6, measured bone growth rate of extant taxa and estimated growth rate of sauropterygians are plotted color-coded on the phylogenetic tree.

## Discussion

### Methodological issues

The extant species used to build the models in our study were taken from previous studies (Cubo et al., 2012; Legendre et al., 2016) with a similar scope and include representatives of all major amniote clades. The models have high statistical significance. Still, the problem, which animals to compare plesiosaurs with, is not easily solved. First, due to their unique body plan, no extant marine counterpart is known. Second, their phylogenetic position and thus their closest living relatives are not unambiguously clear. It might appear obvious to draw comparisons to extant reptiles, preferably marine ones, such as sea turtles. On the other hand, physiologically, plesiosaurs might resemble more endothermic birds and mammals.

A comparison with large aquatic mammals and birds is missing in the current study. Including these taxa might inform about the dependency of vascularity on secondary aquatic adaptations. However, comparisons with modern cetaceans are hardly possible, since their long bones show a spongious organization (de Buffrénil & Schoevaert, 1989; Houssaye, Sander & Klein, 2016). As a result, measurements of primary osteon density are skewed by reorganization of the spongiosa. Within sirenians, the manatees have compact bone, but they are slow swimmers in shallow water, have a low metabolism and do not match plesiosaurs concerning lifestyle and habitat (Houssaye, 2009). Penguins show osteosclerotic humeri with a small medullary cavity and are active swimmers (Meister, 1962; Ksepka et al., 2015). Thus, in future analyses of plesiosaur histology, large-bodied penguin taxa should be included in the predictive models.

Relationships to bone growth rate and metabolism have been described for several histological characteristics. The present study uses primary osteon density, thus, the degree of vascularization, as predictor variable. Only for bone vascularity a dependence on growth rate and metabolism has been proven statistically (de Margerie, Cubo & Castanet, 2002), which is why this variable was chosen for both models. Future studies might analyze the power of alternative variables. We also suggest future studies to include more numerous taxa. This is, however, not easily solved, as bone histology is an invasive method that requires the permission to cut the bones. Especially for rare fossil specimens the method complicates access to material and data acquisition.

Still, for interpreting vascularity, a few problematic aspects have to be taken into account. Besides growth rate and phylogeny, bone type, body size, and thus bone size may also influence vascularity (Montes, Castanet & Cubo, 2010). As bone size increases, vascularity increases, because the blood vessels of the periosteum do not provide sufficient blood supply to the cortex, necessitating cortical bone vascularization. Cubo et al. (2005) provided statistical evidence for the relationship between vascularity and bone size in sauropsids. Houssaye (2013), on the other hand, rejected a connection between vascularity and body size. Concerning bone type, this study investigates three humeri and five femora in fossils species using a model based on femora of extant species. We are aware that bone type might influence the results, however, in plesiosaurs, humeri, and femora serve the same function (same type of movement) and are strikingly similar to each other in both morpholoy and histology. Whereas basal eosauropterygians have a different type of locomotion and shape differences in humeri and femora, the bone histology again is very similar. Since plesiosaurs are in the focus of our study, we included three humeri in combined analyses.

A further problem is that in aquatic taxa, bone vascularity might be influenced by general bmd or the presence of primary cancellous bone as an aquatic adaptation. However, in the areas where histological measurements were taken in this study, all specimens had compact bone.

To quantify vascular density, this study applies a new approach, where vascular area, including lamellar infillings of osteons, is compared to whole bone area. Although measuring vascular density as primary osteon density is currently the most appropriate, future work must find ways to quantify three-dimensional vascular density in order to correctly interpret this parameter and its importance for metabolism and growth.

The results of both analyses, bone growth rate and metabolic rate, show the same pattern among eosauropterygians. This is probably based on the fact that primary osteon density was chosen as a predicting variable in both models and that growth rate and metabolic rate presumably are linked (Case, 1978). Considering the result of Montes et al. (2007), statistically demonstrating a linear positive correlation between bone growth rate and RMR, both analyses cannot be taken as mutually supportive.

The estimated values roughly correlate with bone and body size of the analyzed specimens. Since in mass-based growth models, an increase of growth rate with body weight in different vertebrate clades can be observed (Case, 1978), bone apposition rate might be tied to body mass and bone size. However, in the present study the values for RMR have been corrected for body mass (Cubo et al., 2012). Since the values for RMR and bone growth rate show a similar pattern, the effect of body mass on the results for growth rate can be neglected.

### Plesiosaurian metabolism

The RMRs of the plesiosaurs Elasmosauridae indet., *Rhaeticosaurus*, and *Plesiosaurus* are in the range of birds or even higher. *Cryptoclidus* is the exception within plesiosaurs with a slightly lower rate. However, it is still higher than the mammals in this study. This result is congruent with previous studies and previously accumulating evidence of an active lifestyle and elevated metabolic rates for plesiosaurs based on microanatomy (Wiffen et al., 1995), qualitative histology (discussed in Wintrich et al., 2017), cosmopolitan distribution (Benson, Evans & Druckenmiller, 2012; Benson & Druckenmiller, 2014; Wintrich et al., 2017), indications for parental care (O’Keefe & Chiappe, 2011), and oxygen isotopes (Bernard et al., 2010) and supports the hypothesis of plesiosaurs as fast and sustained swimming, active predators. Also, the growth rates of the plesiosaurs Elasmosauridae indet., *Rhaeticosaurus*, and *Plesiosaurus* are in the range of birds. *Cryptoclidus* shows a slightly lower growth rate, but is still in the range of birds.

However, our results depend on the correct interpretation of the first growth mark, which has been discussed in Wintrich et al. (2017). Wintrich et al. (2017) furthermore estimated maximum plesiosaur bone growth rate by an alternative approach, using bone growth marks in four specimens (three of which were also used in this study), leading to values of 16.6 μm/day in *Plesiosaurus*, 23.2 μm/day in *Rhaeticosaurus*, 36.3 μm/day in *Cryptoclidus*, but also as high as 90.4 μm/day (Plesiosauridae indet, not used in this study). First, these estimations show a high variance, and second, the obtained values are lower on average than the estimations of the present study (60–99.7 μm/day). Anyway, despite the differences in bone growth rate by estimations from growth marks and by modeling in a statistical framework, no overlap of bone growth rate with extant reptiles can be recorded for either set of results. Both analyses place plesiosaur growth rates in the range of mammals and birds, arguing for an endothermic metabolism. This gap between plesiosaurs and extant reptiles is also true concerning calculated RMRs.

Similar to what had been proposed for dinosaurs, an elevated metabolism might have allowed plesiosaurs seasonal migrations to colder environments (Ostrom, 1969; Currie, 1989; Paul, 1997). These habitats may have provided the selective pressure to develop a heat generation mechanism (Schweitzer & Marshall, 2001).

### The evolution of metabolism in sauropterygians

For *Anarosaurus* and *Neusticosaurus* the present study suggests a metabolic rate and bone growth rate similar to or slightly higher than mammals. This would shift the origin of high metabolism back to basal eosauropterygians. For *Neusticosaurus pusillus* and *Neusticosaurus peyeri*, slow-growing lamellar-zonal bone with high compactness had been described previously (Sander, 1989, 1990; Hugi et al., 2011). Klein (2010) described furthermore a low vascular density with dominating longitudinal orientation at midshaft. The here studied *N. edwarsii*, however, grows to larger body size and exhibits a higher degree of vascularization. *Anarosaurus* has fast-growing FLB tissue with a rather high degree of radial vascularization and thus shows a high similarity in histology to *Pistosaurus* and plesiosaurs (Klein, 2010). Thus, a high growth rate was suggested for *Anarosaurus* (Klein, 2010).

The lowest values for metabolic rate and growth rate of all sauropterygians analyzed in this study were obtained for *Nothosaurus*. Previously, a lamellar-zonal bone tissue type with a moderate to low vascular density and longitudinal and radial vascularization had been described (Klein, 2010; Klein et al., 2016). Whereas Klein (2010) hypothesized that nothosaurs grew at rates comparable to reptiles, rare areas of FLB had been detected, leading to the hypothesis that they grew at higher growth rates than previously expected (Klein et al., 2016). This view is supported by our quantitative estimations. On the other hand, exclusive occurrence in the warm shallow Tethys argues against fast sustained swimming and elevated metabolism. Nothosaurs are a very heterogeneous clade (Klein, 2010; Klein et al., 2016), and the selection of specimens might have a great influence on the results.

The estimated bone growth rate for *Pistosaurus* is below the one for three plesiosaurs and above those of more basal eosauropterygians. It is most similar to the rate of *Cryptoclidus* and thus still in the range of birds. The same pattern is also true for RMR in *Pistosaurus*.

The evolution of a high growth rate and fast metabolism in the common ancestor of pistosaurs and plesiosaurs, before the plesiosaur radiation, had already been suggested in previous studies. Krahl, Klein & Sander (2013) demonstrated the presence of FLB in *Pistosaurus*. Further evidence comes from paleobiogeography, with the occurrence of basal pistosauroids (*Augustasaurus*: Sander, Rieppel & Bucher (1997); Rieppel, Sander & Storrs (2002); *Yunguisaurus*: Sato et al. (2010); *Pistosaurus*: Hagdorn & Rieppel (1999)) already indicating a Northern hemisphere and presumably cosmopolitan distribution. A difference between basal pistosauroids and plesiosaurs is found in humerus morphology and thus in locomotion. Whereas the former applied lateral undulation for propulsion, the latter evolved flippers for underwater flight (Carpenter et al., 2010). This partially reflects an increasing adaptation to a pelagic habitat.

Quantitative estimations of growth rate and metabolic rate in the present study are in accordance with histology, geology and lifestyle of *Pistosaurus*.

The facts that FLB is present in several sauropterygian taxa (Klein, 2010; Krahl, Klein & Sander, 2013; Wintrich et al., 2017) and that all analyzed specimens show a metabolic rate and growth rate similar to those of mammals or birds will inevitably lead to the question of their evolutionary origin. Placodonts are the most basal sauropterygians and restricted to the shallow water environment of the Tethys. This habitat is also reflected by bone mass increase (Houssaye, 2009; Klein, 2010; Scheyer et al., 2012; Klein et al., 2016). Some placodonts have fast-growing FLB with a very high degree of vascularization and both longitudinal and radial canals (de Buffrénil & Mazin, 1992; Klein, 2010; Houssaye, 2013; Klein et al., 2016) and may have had an equally high growth rate as pistosauroids. The histology of placodonts implicates that the ability to lay down FLB, and thus that an elevated metabolic rate and growth rate may have evolved at the basis of Sauropterygia or may have even been inherited from terrestrial progenitors.

Oxygen isotope analyses (Bernard et al., 2010) and histology (de Buffrénil & Mazin, 1992) suggest that ichthyosaurs also were able to maintain stable and high body temperatures. Furthermore, among recent species, the leatherback turtle (*D. coriacea*) shows an elevated metabolic rate (although not as high as predicted for plesiosaurs) and a trend towards endothermy as well (reviewed in Wallace & Jones, 2008). If and when other reptile clades independently evolved a metabolic rate higher than in ectothermic taxa, it may have also evolved independently in stem sauropterygians.

### Endothermy in sauropterygians

Endothermy, the capability to generate metabolic heat and keep a constant body temperature, has evolved several times independently. In mammals and birds, exhibiting true endothermy, the heat is produced continuously at rest in internal organs such as brain, heart, liver, kidney, and gut (Else, Turner & Hulbert, 2004; Hulbert & Else, 2004; Clarke & Pörtner, 2010).

However, in the last decade, an impressive increase in studies on thermometabolism of extinct vertebrates and a shift from qualitative observations to quantitative modeling was recorded (Nespolo et al., 2011). For fossil archosauromorph metabolism and the evolution of a high metabolic rate in modern birds, recent statistical analyses estimate for basal forms a higher metabolism than in extant ectotherms and for Mesozoic theropod dinosaurs even a metabolism close to that of modern birds (Legendre et al., 2016). Also in the lineage of synapsids, qualitative studies provided evidence for elevated metabolic rates (Shelton et al., 2012; Shelton & Sander, 2017; Laurin & de Buffrénil, 2016). Recently, elevated metabolic rates of an extinct synapsid were determined for the first time quantitatively (Olivier et al., 2017).

Besides, other taxa, such as tunas and lamnid sharks (Bernal et al., 2001; Donley et al., 2004; Graham & Dickson, 2004) and leatherback turtles (Wallace & Jones, 2008), exhibit homeothermy, that is, the ability to maintain a constant body temperature (Hickman et al., 2001). This can be achieved by constant swimming and subsequent heat production by red muscle tissue. Leatherback turtles have been discussed in terms of thermoregulation by large body size and insulation; a strategy also assumed for sauropod dinosaurs and termed mass homeothermy (Paladino, O’Connor & Spotila, 1990; Grady et al., 2014). Large size alters the surface/volume ratio and subsequently reduces heat loss (Paladino, O’Connor & Spotila, 1990). This adaptation allows leatherback turtles to keep a constant warm body temperature even in cold arctic waters with temperatures down to 0 °C (Frair, Ackman & Mrosovsky, 1972).

Since plesiosaurs are large aquatic reptiles as well, with a body size similar to or larger than leatherbacks, and since they are inferred to have been constantly swimming, a similar thermoregulation as leatherback turtles may explain their lifestyle and habitat as well. However, as plesiosaurians display typical endothermic bone histology with FLB and high vascularization, heat production and maintenance exclusively by locomotion and size can be rejected and true endothermy is suggested further on.

However, additional comparisons with leatherback turtles can be drawn considering the pits on the epiphysial surface of long bones. Like in plesiosaurs, in leatherback turtles and some fossil protostegid turtles, large vascularized cartilage canals enter the cartilage of the epiphysis (transphyseal vascularization; Rhodin, 1985). Transphyseal vascularization is not thought to originate from large size, but from fast growing cartilage and thus from the prerequisite high metabolism (Rhodin, 1985; Liebe & Hurum, 2012).

In plesiosaurs, a pelagic lifestyle remains beyond doubt and the estimated impressively high metabolic rate may have been the prerequisite for this lifestyle. As a consequence, plesiosaurs were only slightly affected by the end-Triassic extinction events with several lineages crossing the Triassic–Jurassic boundary (Wintrich et al., 2017). In contrast, most other marine reptile clades, including basal sauropterygians, went extinct in the Late Triassic, which may be due to their shallow water habitat and their lower (although still high) metabolic rate. Their extinction had already previously been linked to their thermophysiology and the disappearance of the warm waters of the Tethys in the late Middle Triassic (Krahl, Klein & Sander, 2013). A similar pattern is found in ichthyosaurs, where only pelagic parvipelvians survived (Fischer et al., 2014).

A further conclusion that may be drawn from the endothermy hypothesized for plesiosaurs is the necessity for a four-chambered heart, which is present in extant birds, crocodiles and mammals. It allows the separation of oxygenated and deoxygenated blood and is essential during activity to supply organs efficiently with oxygen and maintain high metabolic levels (Schweitzer & Marshall, 2001). We thus suggest the independent evolution of a four-chambered heart in plesiosaurs.

## Conclusion

This work analyzes plesiosaurian metabolism and growth rate for the first time quantitatively by statistical modeling. The PEM approach applied here allows the inclusion of a histological predictor variable (primary osteon density) while taking into account phylogenetic relationships. For plesiosaurs, predicted values for both metabolism and growth rate are in the range of birds. Investigating the evolutionary development of the parameters in the sauropterygian lineage shows that already more basal forms, including pachypleurosaurs, *Nothosaurus*, and *Pistosaurus* have values in the range of mammals or higher. These results provide evidence that plesiosaurs and probably even their ancestral forms had an equally high metabolism as mammals and birds do and, thus, were truly endothermic. Furthermore, the high metabolism allowed them to grow fast by FLB tissue. The results obtained by quantitative bone histology are congruent with earlier assumptions of an elevated metabolism based on histological observations (Wintrich et al., 2017), cosmopolitan distribution, lifestyle (Wiffen et al., 1995), reproductive strategy (O’Keefe & Chiappe, 2011), and isotope analyses (Bernard et al., 2010). The evolution of endothermy would explain the success of plesiosaurs, their survival in the Late Triassic and their subsequent diversification.

## Supplemental Information

10.7717/peerj.4955/supp-1 Supplemental Information 1 Histological measurements, models and sections. Tables of histological measurements of extant and fossil species are given. The parameters of the models to estimate metabolic rate and growth rate are listed. The bone histological sections of the fossil specimens used are displayed.

10.7717/peerj.4955/supp-2 Supplemental Information 2 Phylogenetic tree for the model on bone apposition rate.

10.7717/peerj.4955/supp-3 Supplemental Information 3 Phylogenetic tree for the model on metabolic rate.

## Additional Information and Declarations

### Data Availability

The following information was supplied regarding data availability: All histological sections are stored in the histology collection of the Steinmann Institut für Geologie, Mineralogie und Paläontologie, University of Bonn, Bonn, Germany.
