# Supplementary material for: Quantitative histological models suggest endothermy in plesiosaurs
Source: PeerJ. 2018 Jun 6;6:e4955. doi: 10.7717/peerj.4955 (PMC5994164; doi:10.7717/peerj.4955)
Supplement: Supplemental Information 1 — Tables of histological measurements of extant and fossil species are given. The parameters of the models to estimate metabolic rate and growth rate are listed. The bone histological sections of the fossil specimens used are displayed. [file peerj-06-4955-s001.pdf]

Supplement

Histological measurements

Table S1 Histological measurements for the predictor variable of primary osteon density in extant species. To obtain these values, we reanalyzed sections from Cubo et al. (2012) and Legendre et al. (2016). The measuring approach is explained in the main text. C = Cubo et al. (2012), L = Legendre et al. (2016), CL = Cubo et al. (2012) and Legendre et al. (2016).

	Primary osteon (vascular) density	Previously used in
Anas platyrhynchos	38.3615	CL
Cavia porcellus	25.4434	CL
Chelodina siebenrocki	0	CL
Crocodylus niloticus	15.3459	CL
Varanus exanthematicus	7.6233	CL
Gallus gallus	39.2857	L
Microcebus murinus	10.9231	CL
Podarcis muralis	0	CL
Mus musculus	8.0986	CL
Varanus niloticus	0	CL
Pelodiscus sinensis	0	CL
Pleurodeles walti	0	L
Trachemys scripta	0	CL
Zootoca vivipara	0	L
Dromaius novaehollandiae	58.2051	C
Lacerta vivipara	0	C
Streptopelia decaocto	62.7300	C
Struthio camelus	63.1929	C
Turdus merula	63.5380	C

Table S2 Histological measurements for the predictor variable of primary osteon density in fossil eosauropterygians. The measuring approach is explained in the main text.

	Specimen number	Primary osteon (vascular) density
Anarosaurus heterodontus	Wijk. 06-38fe	29.1428
Neusticosaurus edwardsii	PIMUZ T3455	20.7594
Nothosaurus sp.	IGWH 21	20.4726
Pistosaurus longaevus	SMNS 84825	44.1756
Plesiosaurus dolichodirus	STIPB R90	61.2496
Rhaeticosaurus mertensi	LWL MfN P 64047, PM3	65.4465
Cryptoclidus eurymerus	STIPB R324	40.5865
Elasmosauridae indet.	OMNH MV 85	61.6824

Mass-specific resting metabolic rate

Table S3 R statistics of the model to predict resting metabolic rate. *t value* refers to the t-rest of the respective line in the table. $Pr(>|t|)$ is the p value for the t-test. $V6$, $V12$, $V4$ and $V5$ are the selected vectors of the model.

	Estimate	Std. Error	t value	Pr(> $\|t\|$)
Intercept	-2.60140	0.10986	-23.679	1.08e-08
Vascular density	1.25973	0.05662	22.250	1.76e-08
V6	-2.19179	0.28100	-7.800	5.24e-05
V12	1.85050	0.28680	6.452	0.000198
V4	1.12452	0.29526	3.809	0.005173
V5	-0.88714	0.28021	-3.166	0.013276

The multiple R-squared value of the model for metabolic rate is 0.9851, the adjusted R-squared value is 0.9758.

The F statistic of the overall analysis is 106 on 5 and 8 DF (degrees of freedom) with a p-value of 4.33e-07.

The AICc of the model including vascular (primary osteon) density as a predicting variable is 21.59739. The AICc of the model including phylogeny only is 24.34401.

The steepness parameter was estimated to be $a = 0$.

Table S4 Mass-specific resting metabolic rate (RMR) in mL O_2 h^{-1} $g^{-0.67}$ estimated from bone histology and taking into account phylogenetic relationships using the PEM approach. Lower and upper values correspond to 95 % confidence intervals.

	Lower	RMR	Upper
Anarosaurus heterodontus	3.431286	4.825407	6.785948
Neusticosaurus edwardsii	2.305951	3.200634	4.442441
Nothosaurus sp.	2.309282	3.14758	4.290194
Pistosaurus longaevus	5.452919	8.033297	11.83474
Plesiosaurus dolichodirus	7.186786	12.03066	20.13930
Rhaeticosaurus mertensi	7.86524	13.06124	21.68988
Cryptoclidus eurymerus	4.013786	7.237769	13.05134
Elasmosauridae indet.	6.31384	12.13612	23.32741

Bone growth rate

Table S5 R statistics of the model used to predict bone apposition rate. *t value* refers to the t-test of the respective line in the table. $Pr(>|t|)$ is the p value for the t-test. $V7$, $V14$ and $V5$ are the selected vectors of the model.

	Estimate	Std. Error	t value	Pr(> $\|t\|$)
Intercept	0.15086	0.15154	0.995	0.340895
Vascular density	1.08176	0.05716	18.926	9.65e-10
V7	-1.85088	0.37837	-4.892	0.000478
V14	1.19846	0.38776	3.091	0.010273
V5	0.95188	0.38500	2.472	0.030989

The multiple R-squared value of the model for bone growth rate is 0.9725, the adjusted R-squared value is 0.9624.

The F statistic of the overall analysis is 97.09 on 4 and 11 DF (degrees of freedom) with a p-value of 1.67e-08.

The AICc of the model including vascular (primary osteon) density as a predicting variable is 26.30375. The AICc of the model including phylogeny only is 31.2819.

The steepness parameter was estimated to be $a = 0$.

Table S6 Bone growth rate in μm/day estimated from bone histology and taking into account phylogenetic relationships using the PEM approach. Lower and upper values correspond to 95% confidence intervals.

	Lower	RMR	Upper
Anarosaurus heterodontus	31.51501	42.39142	57.02153
Neusticosaurus edwarsii	22.84954	29.79674	38.85621
Nothosaurus sp.	22.54019	29.37212	38.27485
Pistosaurus longaevus	47.66656	65.66986	90.47287
Plesiosaurus dolichodirus	60.50754	92.89297	142.61200
Rhaeticosaurus mertensi	65.48237	99.68617	151.75600
Cryptoclidus eurymerus	35.91600	60.04474	100.38330
Elasmosauridae indet.	50.07065	93.59182	174.94140

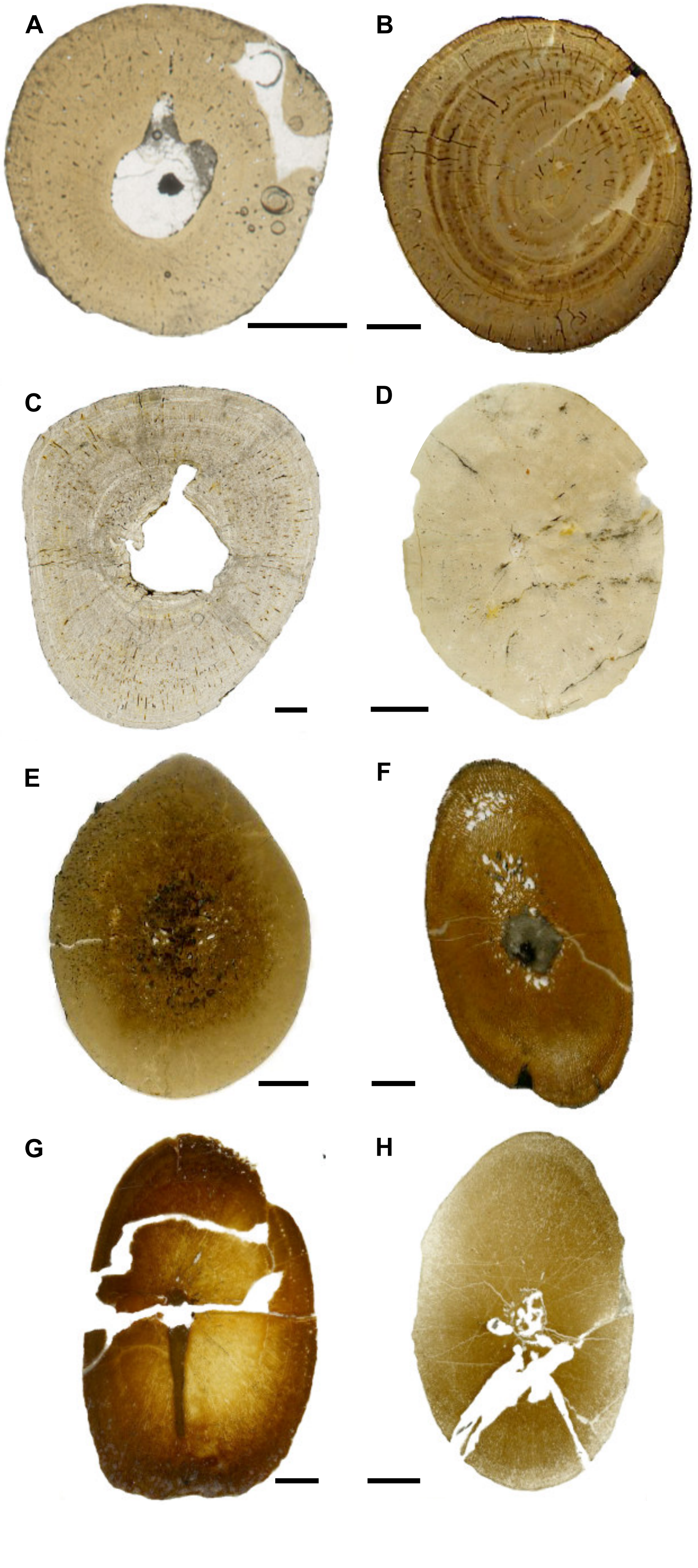
A
B
C
D
E
F
G
H

Figure S1 (Previous page) Bone cross sections analyzed in this study. **A** *Anarosaurus heterodontus* Wijk. 06-38fe, scale bar = 1 mm. **B** *Neusticosaurus edwardsii* PIMUZ T3455, scale bar = 1 mm. **C** *Nothosaurus* sp. IGWH 21, scale bar = 1 mm. **D** *Pistosaurus longaevus* SMNS 84825, scale bar = 5 mm. **E** *Rhaeticosaurus mertensi* LWL MfN P 64047, scale bar = 5 mm. **F** *Plesiosaurus dolichodirus* STIPB R90, scale bar = 5 mm. **G** *Cryptoclidus eurymerus* STIPB R 324, scale bar = 1 cm. **H** Elasmosauridae indet. OMNH MV 85, scale bar = 1 cm. Photos by Corinna V. Fleischle.
